# The effectiveness of automated adjustment of inspired oxygen in preterm infants receiving respiratory support compared with manual: A systematic review and meta‐analysis

**DOI:** 10.1002/pdi3.57

**Published:** 2024-05-22

**Authors:** Yihan Zhang, Yuxuan Du, Yuan Shi

**Affiliations:** ^1^ Department of Neonatology Children’s Hospital of Chongqing Medical University National Clinical Research Center for Child Health and Disorders, Ministry of Education Key Laboratory of Child Development and Disorders Chongqing Key Laboratory of Child Rare Diseases in Infection and Immunity Chongqing Key Laboratory of Pediatrics Chongqing China

**Keywords:** automate, efficacy, meta‐analysis, preterm infants

## Abstract

Preterm infants may need supplemental oxygen due to immature lungs. Regardless of the type of oxygen therapy used, bedside clinicians frequently adjust the FiO_2_ level. Automatic oxygen management is progressively developing as a viable alternative to these corrections. The purpose of this study is to compare the efficacy and safety of automated versus manual oxygen control in preterm infants receiving respiratory support. All the studies were searched from PubMed, Web of Science, Cochrane Library, Embase, CNKI, Wan Fang, VIP, and CBM on 7 May 2022. PICOS criteria were as follows: (P) participants were preterm infants receiving respiratory support; (I) intervention was automatic oxygen control; (C) comparator mode was manual oxygen control; (O) the primary outcome was the percentage of time within the target pulse oxygen saturation range; (S) randomized clinical trials. Sixteen studies were included in this meta‐analysis. The results showed that automated oxygen control can increase the percentage of time spent within the target SpO_2_ range while reducing the percentage of time spent above the target SpO_2_ range, the percentage time of hypoxemia, and manual FiO_2_ adjustments/hour. No significant difference was observed in the percentage of time spent below the target SpO_2_ range, incidence of bradycardia, and the mean SpO_2_ or mean FiO_2_ level. Automatic oxygen control can improve preterm infants' oxygen saturation, reduce periods of hypoxemia and the percentage of time spent above the target SpO_2_ range, and ease the workload of medical staff without affecting the mean FiO_2_ and mean SpO_2_ levels.

## 1 INTRODUCTION

Because their lungs are immature at birth, preterm infants often need supplemental oxygen for prolonged periods. For premature infants, the peripheral oxygen saturation (SpO_2_) level has an immediate influence on their health. Unrestricted, unmonitored oxygen therapy has the potential to cause harm while providing no significant benefit over administered oxygen therapy.^1^ Hyperoxemia or hypoxemia can lead to long‐term complications in newborns, such as chronic lung disease, retinopathy of prematurity (ROP), brain tissue damage, and even life‐threatening complications.^2^, ^3^ The oxygen concentration inhaled by preterm infants is usually strictly controlled through manual adjustments made by medical staff. Recommended by the American Academy of Pediatrics, in a number of countries, a nurse‐to‐patient ratio of 1–3 or 4 is needed for infants with the lowest acuity levels.^4^ However, in actual clinical work, the number of preterm infants is far greater than that of bedside clinicians. This increases the workload of bedside clinicians and easily exposes preterm infants to blood oxygen saturation levels outside the target range, increasing their risk of long‐term complications despite clinicians' best efforts. In some studies, SpO_2_ targets were found to be challenging: a high percentage of the time was spent outside the target range, and there were often prolonged episodes of hypoxemia and hyperoxemia.^5^, ^6^, ^7^

To solve these problems, an automatic control system for FiO_2_ was developed. Some neonatal intensive care units worldwide have begun to utilize computer algorithms to modulate FiO_2_ automatically to achieve more effective SpO_2_ targeting and the benefits that may follow.^8^ Although the automation system seems superior in concept, clinicians have raised concerns about whether the current form of the algorithm can effectively solve the problems. For instance, whether the monitoring of the neonatal oxygenation status is accurate, whether the algorithm for automatic oxygen control is appropriate, whether the increase in the time spent within the target SpO_2_ range effectively improves the prognosis, etc., are all the concerns.^9^ A meta‐analysis of the relevant directions was previously made available. This article is a supplement and an update for this previous meta‐analysis.^8^ In this systematic review with a meta‐analysis of clinical trials, we explored the efficacy and safety of automated oxygen control compared to manual oxygen control in preterm infants receiving respiratory support.

## 2 MATERIALS AND METHODS

This meta‐analysis was conducted based on the preferred reporting items for systematic reviews and meta‐analyses guidelines (29 March 2021).^10^ The study protocol has been registered in the International Prospective Register of Systematic Reviews database (ID: CRD42022306176).

### 2.1 Eligibility criteria

PICOS criteria were as follows: (P) participants were preterm infants receiving respiratory support; (I) intervention was automatic oxygen control; (C) comparator mode was manual oxygen control; and (O) the primary outcome was the percentage of time within the target pulse oxygen saturation (SpO_2_)range; the secondary outcomes were the percentage of time below and above the target SpO_2_ range, the percentage of hypoxemia, bradycardia events per 24 h (hereinafter referred to as bradycardia), manual FiO_2_ adjustments per hour, and mean FiO_2_ and SpO_2_; and (S) only randomized clinical trials (RCTs). We excluded cohort studies, case‐control studies, single‐arm studies, case reports, editorials, and letters. Publications are limited to the English language or Chinese.

### 2.2 Search strategy

Firstly, two authors separately searched Medical Subject Headings for all the terms of “oxygen,” “automate,” and “infant.” Then we combined the terms by the Boolean logic, searching eight databases: PubMed, Web of Science, Cochrane Library, Embase, CNKI, Wan Fang, VIP, and CBM with “(((((Oxygen) OR (Oxygen‐16)) OR (Oxygen 16)) OR (Dioxygen)) AND ((((Automated) OR (Automatic)) OR (closed loop)) OR (closed‐loop))) AND ((((((Infant, Newborn or Newborn Infant) OR (Newborn Infants)) OR (Newborns)) OR (Newborn)) OR (Neonate)) OR (Neonates))” as keywords on 7 May 2022. In addition, some articles were searched by manually reading literature.

### 2.3 Quality assessment

All included trials were assessed by using the RoB2 (Version 2 of the Cochrane tool for assessing risk of bias in randomized trial) version dated 15 March 2019, which contains the following domains: “Randomization process,” “Deviations from intended interventions,” “Missing outcome date,” “Measurement of the outcome,” “Selection of the reported result,” and “Overall Bias.” RoB of RCTs can be judged with “low risk of bias,” “some concerns,” or “high risk of bias.” Two authors (ZYH and DYX) assessed study quality independently, and disagreements were resolved by consensus.

### 2.4 Data extraction

We used Microsoft Excel to construct a data extraction form. Items to be extracted include author, publication year, country; study design, method of intervention, control group, experience group, and sample size; patient characteristics including, gestational age, gestational weight, age at study, and weight at study; outcome including the percentage of time within/below/above the target SpO_2_ range, the percentage of hypoxemia, bradycardia events per 24 h (hereinafter referred to as bradycardia), manual FiO_2_ adjustments per hour, and mean FiO_2_ and SpO_2_.

Additionally, if the outcomes we need were only available as median, maximum, minimum, or quartiles, we would use mean–variance estimation (https://www.math.hkbu.edu.hk/~tongt/papers/median2mean.html) to calculate the mean and standard deviation.^11^, ^12^, ^13^, ^14^

### 2.5 Study records

All search records were imported to NoteExpress software. Two reviewers (ZYH and DYX) independently extracted data and assessed the trial methodology by thoroughly reading the abstracts and full text of the studies that met our inclusion criteria. In cases of differences of opinion during the study selection process, a third author (SY) was consulted.

### 2.6 Data synthesis and analysis

All analyses were performed by using Review Manager (RevMan) version 5.4 (Copenhagen: The Nordic Cochrane Centre, The Cochrane Collaboration, 2014) and STATA software (version 14.0; StataCorp LP). Mean difference (MD) with 95% confidence intervals (95% CI) was used for continuous data. We used *I* ^2^ to evaluate the heterogeneity of included trials. If *I* ^2^ was greater than 50%, it indicated high heterogeneity. When it revealed high heterogeneity, random effect models would be used to synthesize data. Otherwise, we would use the fixed‐effect model. Publication bias was assessed by funnel plots.

### 2.7 Subgroup analysis

Based on the range of target oxygen saturation included in the study and a previous meta‐analysis, the outcomes were performed in two groups: the target SpO_2_ ranges between 90%–96% and 85%–96%, respectively.

### 2.8 Sensitivity analysis

We achieved sensitivity analysis by removing included studies one by one and then merging the effect quantity. The results that changed significantly after elimination would be clarified in this study.

### 2.9 Publication bias

Publication bias of included trials was assessed using funnel plots in RevMan 5.3. If the included trials had no bias, the points on the funnel plot were spread out symmetrically around the estimated true value of each independent study effect point, showing an inverted symmetric funnel shape. If bias was present, an asymmetric funnel plot was presented, with the more pronounced asymmetry suggesting a greater degree of bias and possible overestimation of the treatment effect.

### 2.10 Certainty of evidence

We used the GRADE (Grading of Recommendations, Assessment, Development and Evaluations) criteria to evaluate the study.

## 3 RESULTS

### 3.1 Study inclusion and characteristics

**FIGURE 1:**
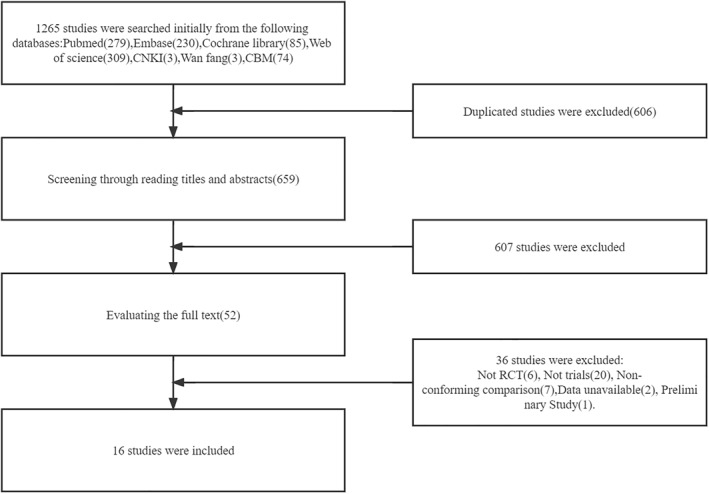
Flow chart of literature search.

A total of 1265 articles were retrieved, and 659 articles remained after removing duplicates. By reading the titles and abstracts, we excluded 607 trials that did not meet our predefined PICOS criteria, while the remaining 52 articles were evaluated by reading the full text (shown in Figure 1). Ultimately, 16 trials were included in this systematic review and meta‐analysis, enrolling 431 participants. Characteristics of included trials are summarized in Table 1.

**TABLE 1:** Characteristics of 16 studies.

Author	Year	Patients	Country	Ventilator or algorithm	Study design	SpO_2_ target range	Research time	Gestational age (week)	Age (day)
Dargaville	2022	35	Australia	VDL1.1 algorithm	A randomized crossover study, single center	90%–94%	24 h	27 (26–28)^a^	17 (12–23)^a^
Dijkman	2021	27	Netherlands	Optiflow interface (Fisher and Paykel Healthcare) with automated FiO_2_ control	A randomized crossover study, single center	88%–95%	24 h	27 + 6 (26+1–29 + 0)^a^	31 (23–42)^a^
Schwarz	2020	19	Germany	Closed‐loop automatic oxygen control	A randomized crossover study, two center	90%–95%	8 h	27 ± 2^b^	24 ± 10^b^
Reynolds	2019	30	UK	A device (IntellO_2_, Vapotherm) using a modified closed‐loop control algorithm	A randomized crossover study, two center	90%–95% 85%–93% 85%–96%	24 h	26 (24–27)^a^	29 (18–53)^a^
Gajdos	2019	12	Germany	Ventilator (Sophie respirator, Fritz Stephan GmbH Medizintechnik GmbH)	A randomized crossover study, single center	88%–96%	24 h	25 (23–26)^a^	31.5 (12–62)^a^
van den Heuvel	2018	41	Netherland	Avea ventilator (CareFusion)	A randomized crossover study, single center	86%–94%	24 h	26 (25–27)^a^	21 (12–29)^a^
van Kaam	2015	80	Netherlands	Avea ventilator (CareFusion)	A randomized crossover study, multicenter	91%–95% 89%–93%	24 h	26 (25–28)^a^	18 (10–29)^a^
Lal	2015	27	UK	Avea ventilator (CareFusion)	A randomized crossover study, single center	90%–95%	12 h	25 (24–27)^a^	16 (9–27)^a^
Wilinska	2015	21	Poland	Avea ventilator (CareFusion)	A randomized crossover study, single center	87%–93%	2.5 h	27 (24–36)^c^	8 (3–23)^c^
Waitz	2015	15	Germany	Avea ventilator (CareFusion)	A randomized crossover study, single center	88%–96%	24 h	25 (23–28)^c^	34 (19–74)^c^
Clarke	2015	16	Australia	Hypoxia algorithm	A randomized crossover study, single center	88%–92%	4 h	26.7 ± 1.3^b^	NA
Hallenberger	2014	34	Germany	Ventilator (Leoni plus; Heinen & Loewenstein GmbH, Bad Ems) with FIO_2_C software	A randomized crossover study, multicenter	85%–94%	24 h	26.4 (23.0–35.3)^c^	29.9 (26.0–35.6)^c^
Claure	2011	32	USA	Avea ventilator (CareFusion)	A randomized crossover study, multicenter	87%–93%	24 h	25 (24–27)^a^	27 (17–36)^a^
Claure	2009	16	USA	Ventilator (Avea, Viasys Healthcare)	A randomized crossover study, single center	88%–95%	4 h	24.9 ± 1.4^b^	33 ± 15^b^
Urschitz	2004	12	Germany	A neonatal FiO_2_ controller established by the authors	A randomized crossover study, single center	87%–96%	24 h	25.5 (24–33)^c^	20.5 (4–78)^c^
Claure	2001	14	USA	A neonatal mechanical ventilator (Babylog 8000, Draeger)	A randomized controlled study, single center	88%–96%	2 h	25 ± 1.6^b^	26 ± 11^b^

*Note*: NA: the age results of this study were presented in a different way than other studies: postmenstrual age of 30.5 ± 2.4 weeks (mean ± SD).

^a^ The results are presented in the median (IQR) format.

^b^ The results are presented in the form of mean ± SD.

^c^ The results are presented in the form of median (range).

### 3.2 Rob of included trials

**FIGURE 2:**
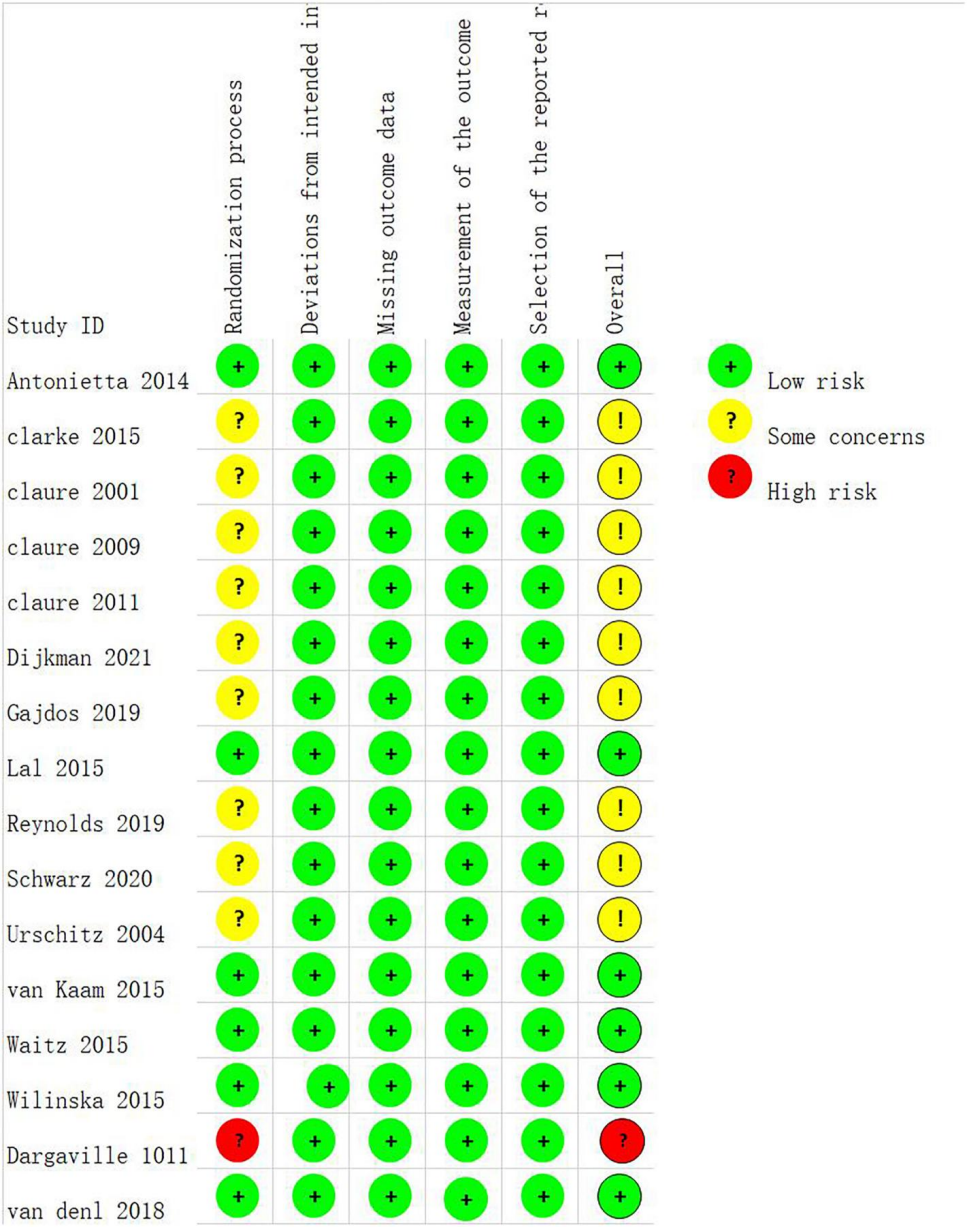
Quality assessment of randomized clinical trials.

We assessed the risk of bias of included trials by using the RoB2. Evaluation domains included bias during randomization, bias for deviation from established interventions, bias for missing outcome data, bias for outcome measures, and bias for selective reporting of outcomes. The trials that did not specify the specific classification method would be treated with “some concerns” (as shown in Figure 2).

## 4 PRIMARY OUTCOME

**FIGURE 3:**
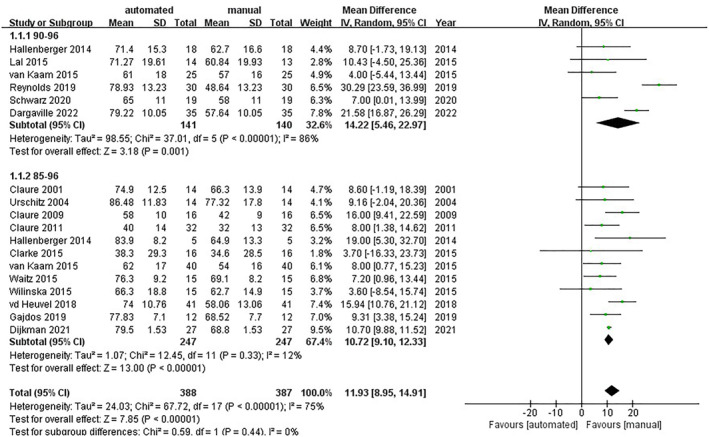
Forest plot of mean difference (MD) of the percentage of time within the target SpO_2_.

The percentage of time within the target SpO_2_ range was presented in all 16 RCTs. The pooled data showed that automatic oxygen control could significantly increase the percentage of time within the target SpO_2_ range (MD = 11.93; 95% CI [8.95, 14.91]; and *I* ^2^ = 75%). Subgroup analysis was as follows: In the group of the SpO_2_ target range from 85% to 96%, the outcome was homogenous (MD = 10.72; 95% CI [9.10, 12.33]; and *I* ^2^ = 12%).^15^, ^16^, ^17^, ^18^, ^19^, ^20^, ^21^, ^22^, ^23^, ^24^, ^25^, ^26^ In the group of the SpO_2_ target range from 90% to 96%, it showed considerable heterogeneity (MD = 14.22; 95% CI [5.46, 22.97]; and *I* ^2^ = 86%)^18^, ^21^, ^27^, ^28^, ^29^, ^30^ (shown in Figure 3).

## 5 SECONDARY OUTCOMES

### 5.1 Percentage of time above the target SpO_2_ range

Thirteen trials showed that automatic oxygen control could decrease the percentage of time above the target SpO_2_ range compared with the manual (MD = −7.15; 95% CI [−10.41, −3.90]; *I* ^2^ = 87%).

In the group of SpO_2_ target range from 85% to 96%, the outcome of automatic control is reported in 10 trials^15^, ^16^, ^17^, ^18^, ^20^, ^22^, ^23^, ^25^, ^26^ (MD = −7.80; 95% CI [−11.76, −3.83]; and *I* ^2^ = 88%).

**FIGURE 4:**
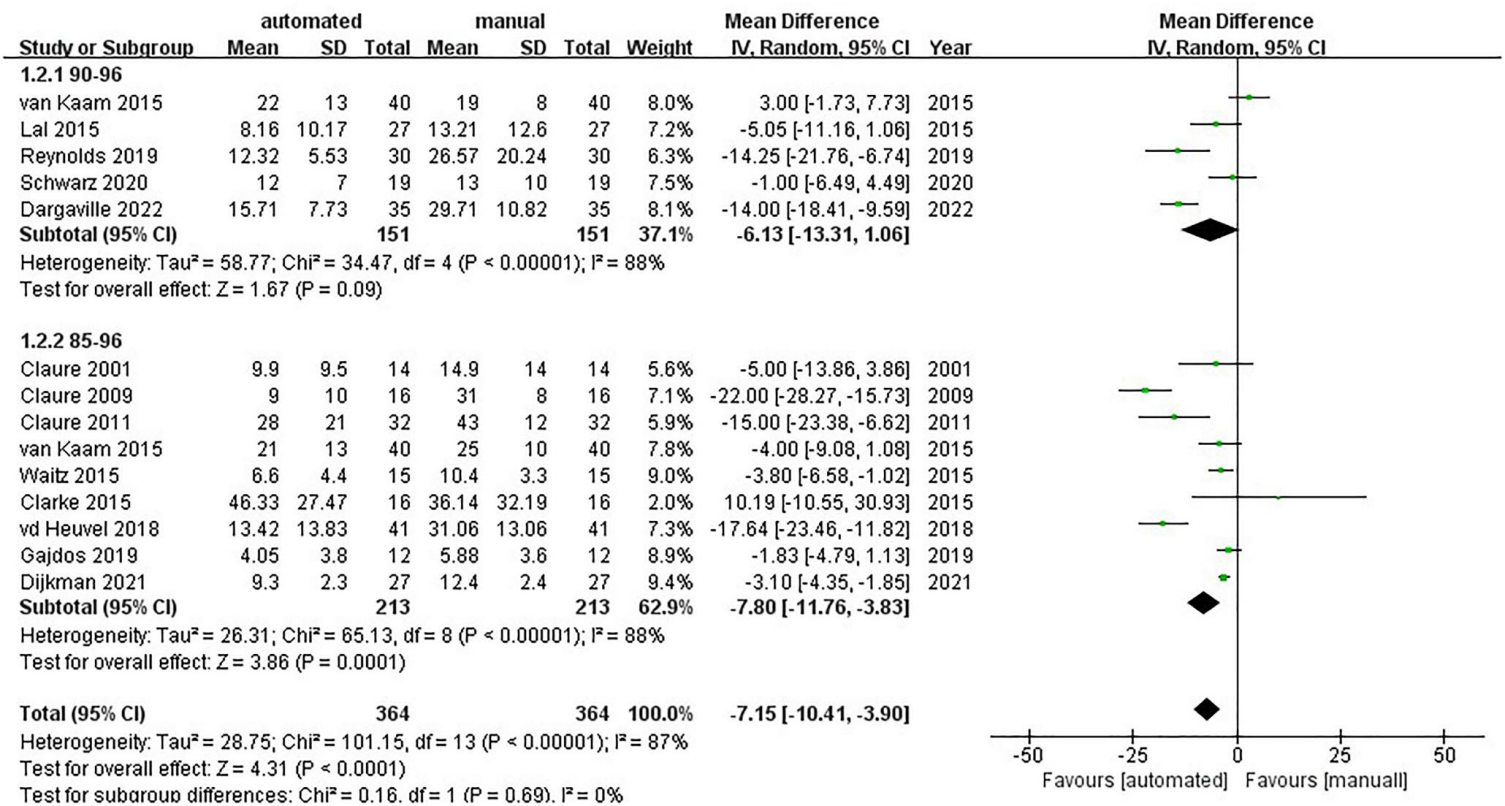
Forest plot of mean difference (MD) of the percentage of time above the target SpO_2_ range.

In the group of SpO_2_ target range from 90% to 96% the outcome of automatic control is reported in 5 trials^18^, ^27^, ^28^, ^29^, ^30^ (MD = −6.13; 95% CI [−13.31, −1.06]; and *I* ^2^ = 88%). However, in the process of sensitivity analysis, the two ways become different after removing the trial of van Kaam^18^ (MD = −8.51; 95% CI [−15.26, −1.76]; *I* ^2^ = 82%) (shown in Figure 4) (Supplementary Material S1).

### 5.2 Percentage of time below the target SpO_2_ range

Thirteen trials presented the outcome of the percentage of time below the target SpO_2_ (MD = −3.12 95% CI [−6.07, −0.17]; *I* ^2^ = 79%).

In the group of the SpO_2_ target range from 85% to 96%, 9 trials reported the percentage of time below the target SpO_2_ range (MD = −0.59; 95% CI [−4.22, 3.04]; *I* ^2^ = 74%).^15^, ^16^, ^17^, ^18^, ^20^, ^22^, ^23^, ^25^, ^26^

**FIGURE 5:**
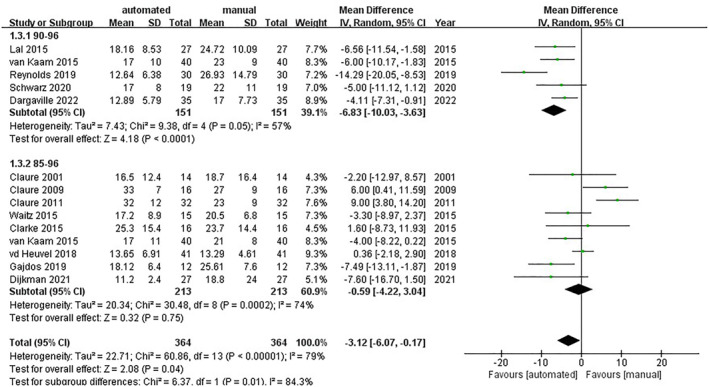
Forest plot of mean difference (MD) of the percentage of time below the target SpO_2_ range.

In the group of the SpO_2_ target range from 90% to 96%, 5 trials reported the percentage of time below the target SpO_2_ range (MD = −6.83; 95% CI [−10.03, −3.63]; *I* ^2^ = 57%)^18^, ^27^, ^28^, ^29^, ^30^ (shown in Figure 5).

### 5.3 Percentage time of hypoxemia

Ten trials included the percentage time when SpO_2_ was less than 80% and showed that automated oxygen control could lower the time of hypoxemia (MD = −0.99; 95% CI [−1.49, −0.50]; *I* ^2^ = 78%).

In the group of the SpO_2_ target range from 85% to 96%, there were a total of seven tests that proved that automated control could reduce the percentage time of hypoxemia with low heterogeneity (MD = −0.95; 95% CI [−1.17, −0.73]; *I* ^2^ = 0%).^15^, ^16^, ^17^, ^18^, ^19^, ^20^, ^22^

**FIGURE 6:**
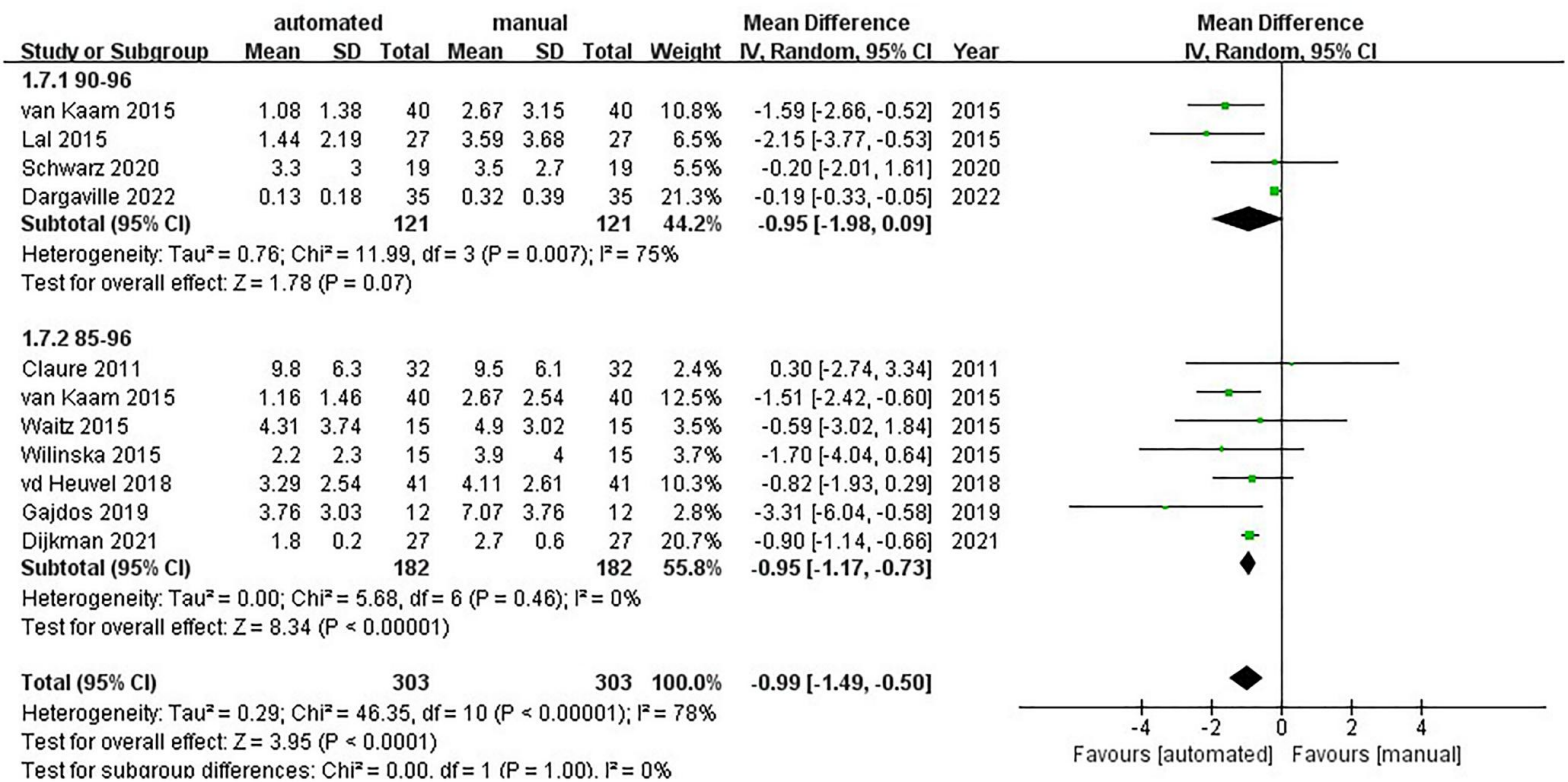
Forest plot of mean difference (MD) of the percentage time of hypoxemia.

In the group of the SpO_2_ target range from 90% to 96%, four trials showed no clear superiority of automatic oxygen control (MD = −0.95; 95% CI [−1.98, 0.09]; *I* ^2^ = 75%).^18^, ^27^, ^28^, ^30^ After excluding one trial [27], the results suggested that automatic oxygen control worked better with reducing heterogeneity (MD = −1.43; 95% CI [−2.38, −0.48]; and *I* ^2^ = 23%) (shown in Figure 6) (Supplementary Material S2).

### 5.4 Manual FiO_2_ adjustments/hour

Different trials have different statistical methods regarding manual FiO_2_ adjustments. For the purpose of analysis, we standardized them by counting the number of adjustments in each hour. Seven trials [5, 9–11, 15, 16, and 20] reported sufficient data to compare manual FiO_2_ adjustments per hour between automated oxygen control and manual oxygen control (SMD = −1.77; 95% CI [−2.52, −1.02]; and *I* ^2^ = 90%) (Supplementary Material S3).

### 5.5 Mean FiO_2_

Ten trials showed the outcome of mean FiO_2_. The trial of Dijkman^15^ was excluded because of its extreme weight. The combined statistics of the remaining trials were as follows: MD = −0.01; 95% CI [−0.02, 0.01]; and *I* ^2^ = 0%^16^, ^17^, ^18^, ^19^, ^20^, ^22^, ^25^, ^26^, ^30^ (Supplementary Material S4).

### 5.6 Mean SpO_2_

The outcome of mean SpO_2_ was presented in 9 trials, showing there was no difference between the two methods (SMD = −0.10; 95% CI [−0.42, 0.23]; *I* ^2^ = 55%)^15^, ^16^, ^17^, ^18^, ^19^, ^20^, ^24^, ^25^ (Supplementary Material S5).

### 5.7 Bradycardia

There were four trials showing that automatic oxygen control did not reduce the risk of bradycardia with homogenous results (MD = −0.76; 95% CI [−2.75, 1.22]; *I* ^2^ = 0%)^16^, ^20^, ^22^, ^23^ (Supplementary Material S6).

### 5.8 Sensitivity analysis

This outcome of the percentage time of hypoxemia reversing after sensitivity analysis was not robust and needed to be cautious. The other results remained stable, and there was no significant difference in heterogeneity after heterogeneity analysis.

### 5.9 Publication bias

We evaluated the publication bias of all the outcomes, and no significant publication bias was found. The funnel plot of the primary outcome can be found in Supplementary Material S7.

### 5.10 Certainty of evidence

The results on GRADE ratings are available in Supplementary Material S8.

## 6 DISCUSSION

In this meta‐analysis, 16 studies including 431 infants were evaluated to compare the effectiveness of automated versus manual FiO_2_ control based on SpO_2_.

This analysis showed that preterm infants managed with automated control spent more time in the predefined target SpO_2_ range compared to those managed with manual control and spent less time with oxygen saturation levels outside of the target range. The improvement was mainly due to a comparable reduction in periods above the target range, especially in the group with an SpO_2_ level of 85%–96% and more consistent results. The reduction in the incidence of hypoxemia and hyperoxemia was an important advantage of automated control, which represented the greatest potential for a lasting clinical benefit of automated oxygen control. The relationship between long‐lasting hyperoxemia and ROP is well established,^31^ and the prevention of hyperoxemia appears to reduce the incidence of ROP. In the same light, hypoxemia appeared to be an important factor in the risk of adverse outcomes in preterm infants.^32^, ^33^ Because there are few studies of short‐term or long‐term prognosis outcomes, the effect of automatic oxygen control on these adverse prognoses needs more targeted research.

Automatic oxygen control showed divergence in the outcome of the percentage of time spent with an SpO_2_ level less than the intended range. In the group with a target SpO_2_ ranging from 90% to 96%, automatic oxygen control reduced the percentage of time spent with an SpO_2_ level below the target range. In the 85%–96% group, there was no difference between the two measurements. This result was different from previous meta‐analyses. Some past studies have shown that this was mainly caused by a higher frequency of episodes with an SpO_2_ level <88%. However, this was not caused by an increase in the duration of hypoxemia. These episodes were often triggered by spontaneous changes in ventilation, and a higher basal SpO_2_ level could attenuate these.^23^, ^34^, ^35^, ^36^ The results of our article were consistent with these findings.

There were far fewer manual FiO_2_ adjustments required during the automated oxygen control period compared with the manual control period. It could not only effectively reduce the workload of bedside clinicians but also alleviate the lack of medical resources. Nevertheless, automatic control could not entirely replace labor, and a certain number of skilled medical staff were still needed. When hypoxemia or hyperoxemia occurred, observation of the ventilator settings and clinical evaluation of the infants were essential, even during automated FiO_2_ control, to identify the individualized etiologies and to implement appropriate interventions.

From the results, we found that most of the outcomes showed high heterogeneity, such as the percentage of time spent above the target SpO_2_ range and the percentage of time spent below the target SpO_2_ range. We speculated the following reasons: 1. Table 1 showed that the equipment used varied from study to study. The rhythms of some studies were even established by the investigators themselves according to their needs. This leads to differences in the adjustment logic, speed, or amplitude of FiO_2_, thus increasing heterogeneity; 2. Pulse oximetry (SpO_2_) is commonly used for monitoring the oxygenation status of neonates for continuous noninvasive monitoring of oxygen delivery to tissues. However, different studies use different measurement intervals; for example, Claure (2011) set the average interval to 2 s in his study, while in Reynolds' study, this interval was set to 8 s. This difference affects the estimation of the duration of hypoxemia (by interpreting a cluster of desaturations as a single prolonged event) and thus the heterogeneity; 3. In the group with an SpO_2_ level ranging from 85% to 96%, the heterogeneity of the primary outcome was significantly lower than that in the overall comparison (*I* ^2^ = 75%→12%). This would seem to suggest higher heterogeneity among studies with broader target ranges. Future studies should consider the effect of such factors on experimental results.

Compared with the previous meta‐analysis,^8^, ^37^, ^38^ there were some differences in the inclusion of studies and some of the outcomes. Firstly, we updated the included literature to include a number of newly published controlled trials and exclude two studies included in previous meta‐analyses because they did not meet the inclusion criteria. Secondly, as mentioned above, in the secondary outcome, we came to a different conclusion. Finally, we have included two new results that automatic oxygen control could maintain an SpO_2_ level in the target range for more time without affecting the mean FiO_2_ and SpO_2_ levels, preventing iatrogenic or unnecessarily wide adjustments in FiO_2_ and SpO_2_.

It is noteworthy that the impact of improved adherence to the target SpO_2_ range on the clinical outcomes did not remain clear^39^ and requires RCTs.^40^

It is essential to note that there are still some limitations of this study. For example, there was no discussion of the algorithms/models of automated oxygen control in this paper. There was also no comparison as to which of the wider or narrower target oxygen saturation targets was more appropriate for preterm infants. Another possible shortcoming was the small size of most studies. More clinical studies on these factors will hopefully be available in future studies.

## 7 CONCLUSION

Automatic oxygen control can improve preterm infants' oxygen saturation, reduce periods of hypoxemia and the percentage of time spent above the target SpO_2_ range, and ease the workload of medical staff without affecting the mean FiO_2_ and mean SpO_2_ levels.

## AUTHOR CONTRIBUTIONS

Yihan Zhang and Yuxuan Du conceived and designed the study, collected and analyzed the data. Yihan Zhang wrote the first manuscript draft. Yuan Shi was the guarantor and revised it critically for important intellectual content.

## CONFLICT OF INTEREST STATEMENT

Yuan Shi is the Deputy Editor‐in‐Chief of Pediatric Discovery. To minimize bias, he was excluded from all editorial decision‐making related to the acceptance of this article for publication. The remaining authors declare no conflict of interest.

## ETHICS STATEMENT

An ethics statement was not required for this study type; no human or animal subjects or materials were used.

## Supporting information

Supplementary Data S1

Figure S1

Figure S2

Figure S3

Figure S4

Figure S5

Figure S6

Figure S7

Figure S8

## Data Availability

All data generated or analyzed during this study are included in this article. Further inquiries can be directed to the corresponding author.
