# Supplementary material for: The effectiveness of automated adjustment of inspired oxygen in preterm infants receiving respiratory support compared with manual: A systematic review and meta‐analysis
Source: Pediatr Discov. 2024 May 22;2(4):e57. doi: 10.1002/pdi3.57 (PMC12118224; doi:10.1002/pdi3.57)
Supplement: Supplementary file 1 — Supplementary Data S1 [file PDI3-2-e57-s008.docx]

Supplementary material 1 Sensitivity analysis of the outcome of the percentage of time above the target SpO2 range

Supplementary material 2 Sensitivity analysis of the outcome of the percentage time of hypoxia

Supplementary material 3 Forest plot of Std. mean difference (SMD) of Manual FiO2 adjustments/hour

Supplementary material 4 Forest plot of mean difference (MD) of mean FiO2

Supplementary material 5 Forest plot of Std. mean difference (SMD) of mean SpO2

Supplementary material 6 Forest plot of mean difference (MD) of bradycardia

Supplementary material 7 Funnel plot of the primary outcome

Supplementary material 8 GRADE grading of the study
