# Supplementary figures and images for: The effectiveness of automated adjustment of inspired oxygen in preterm infants receiving respiratory support compared with manual: A systematic review and meta‐analysis
Source: Pediatr Discov. 2024 May 22;2(4):e57. doi: 10.1002/pdi3.57 (PMC12118224; doi:10.1002/pdi3.57)

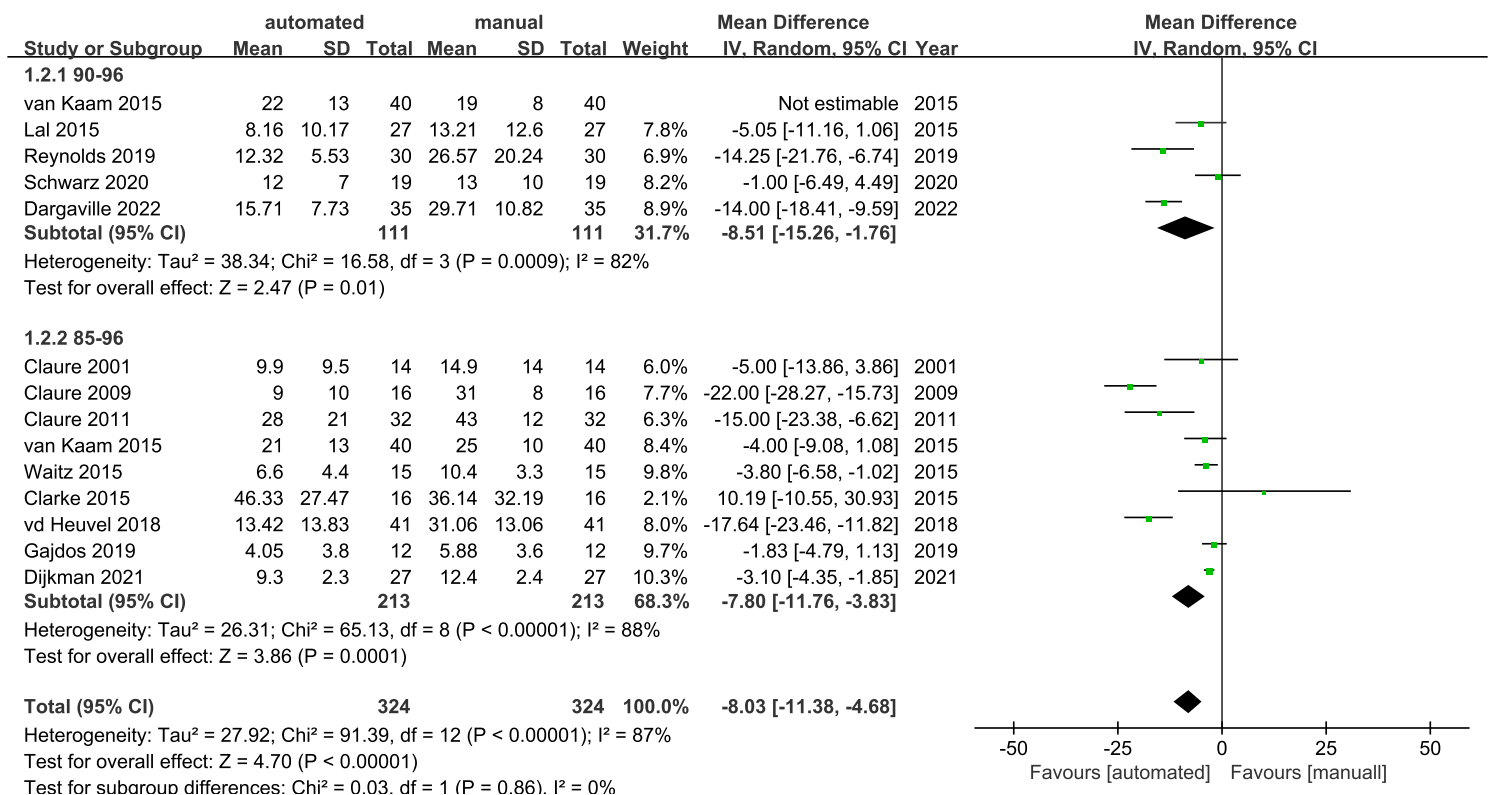

Supplement: Supplementary file 2 — Figure S1 [file PDI3-2-e57-s001.pdf]

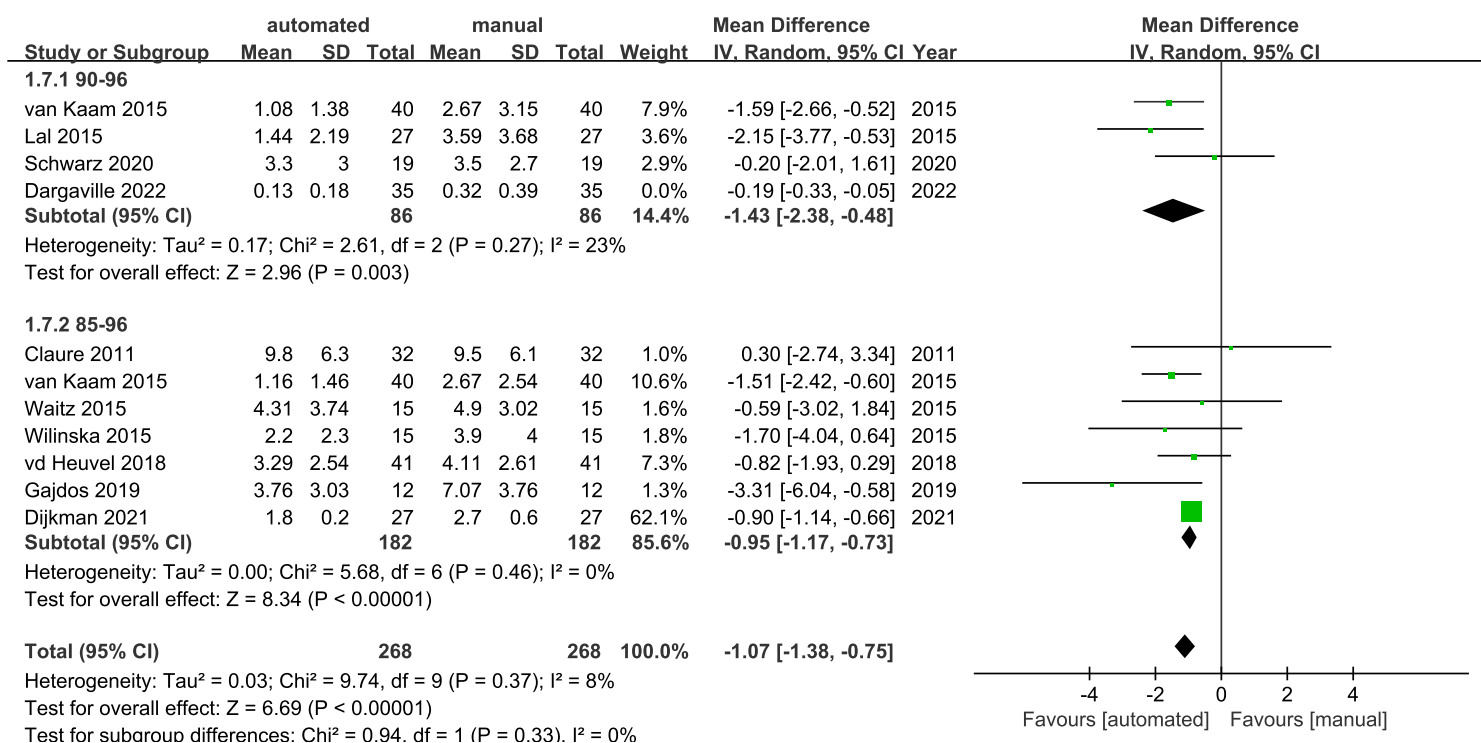

Supplement: Supplementary file 3 — Figure S2 [file PDI3-2-e57-s009.pdf]

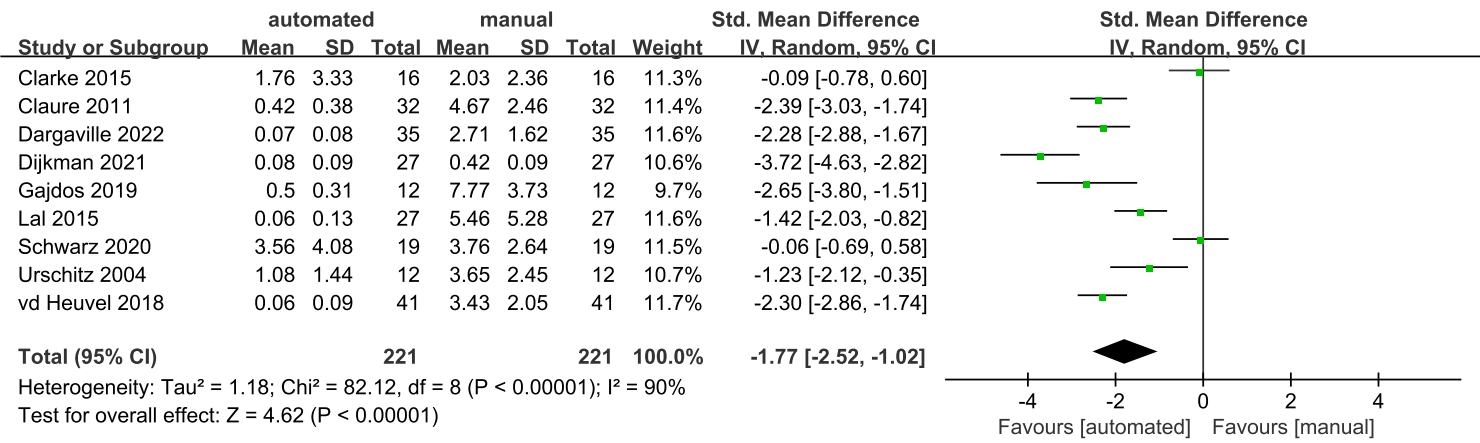

Supplement: Supplementary file 4 — Figure S3 [file PDI3-2-e57-s005.pdf]

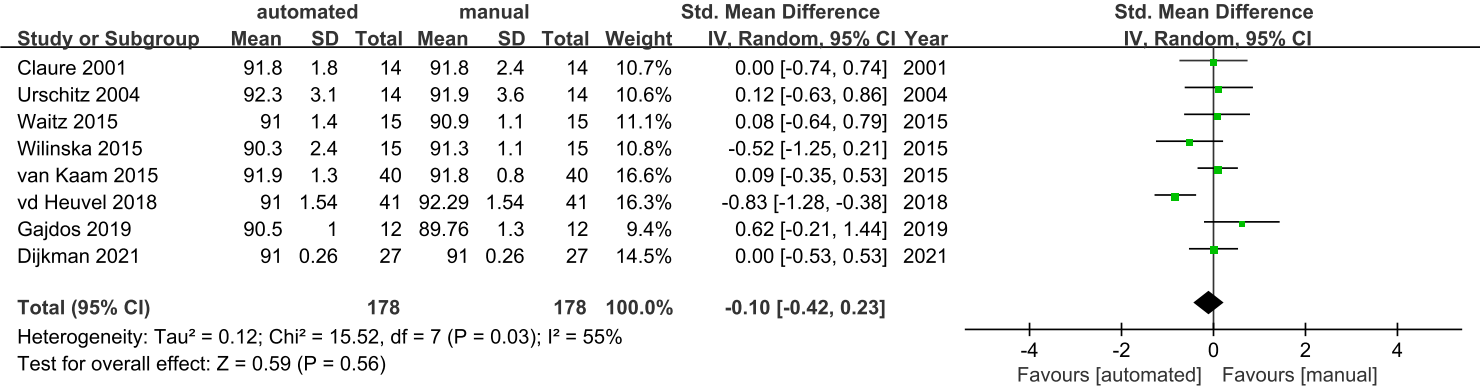

Supplement: Supplementary file 6 — Figure S5 [file PDI3-2-e57-s002.pdf]

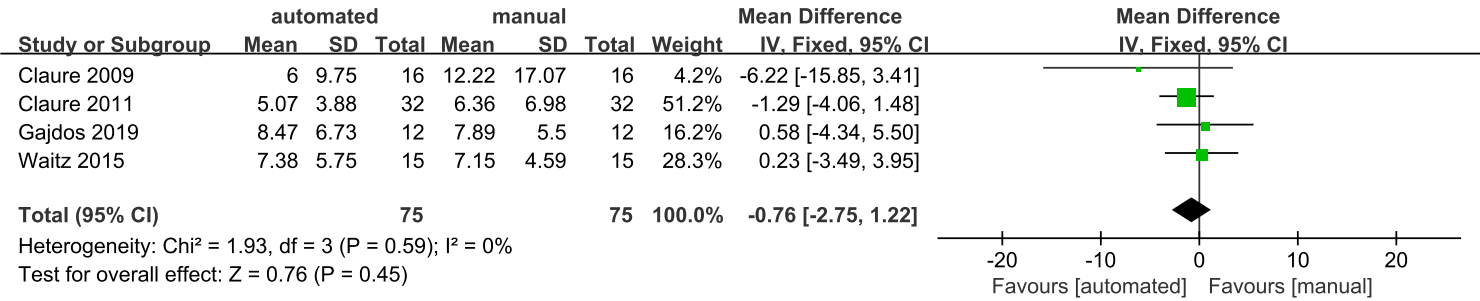

Supplement: Supplementary file 7 — Figure S6 [file PDI3-2-e57-s003.pdf]

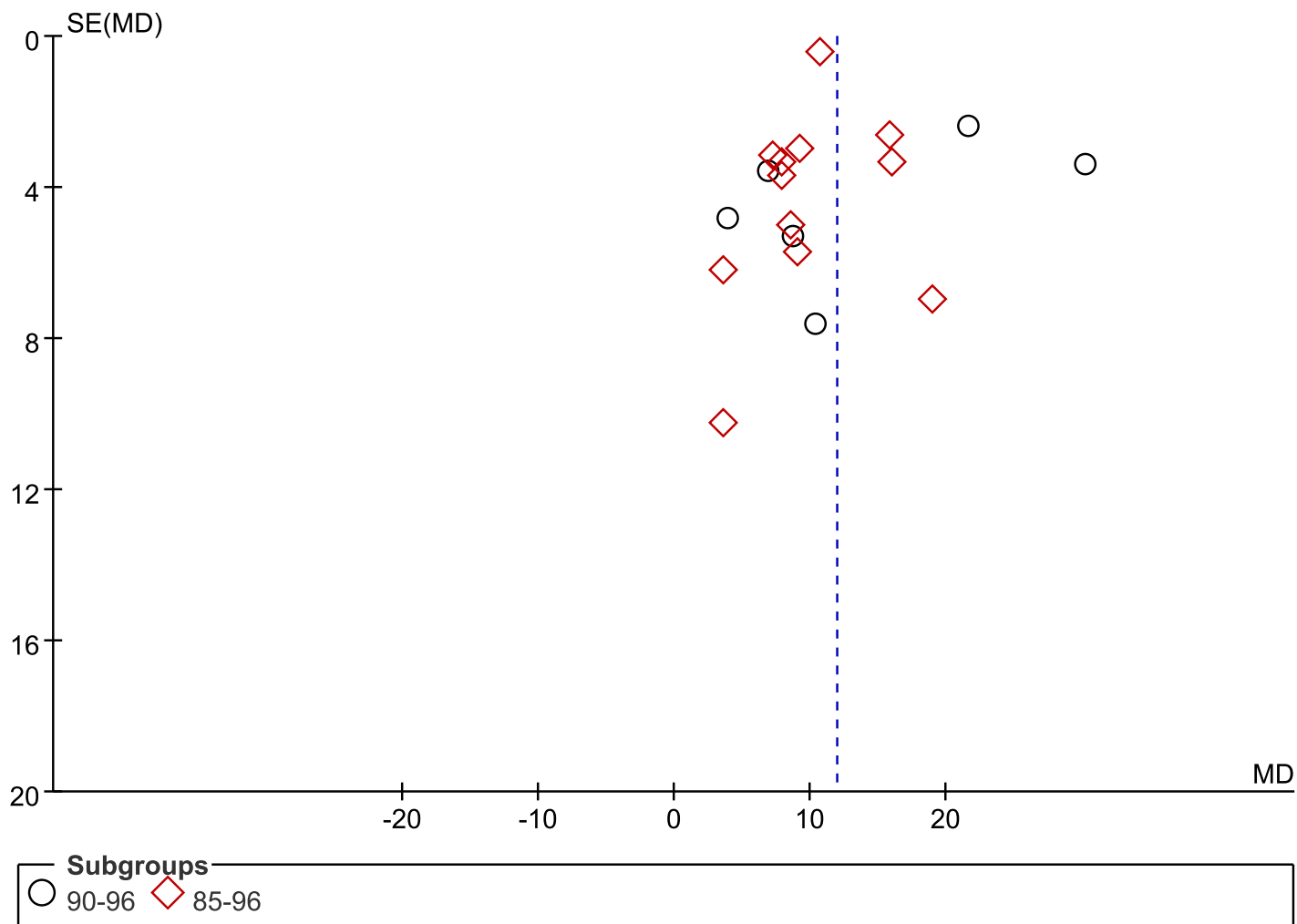

Supplement: Supplementary file 8 — Figure S7 [file PDI3-2-e57-s006.pdf]
