# Supplementary material for: The effectiveness of automated adjustment of inspired oxygen in preterm infants receiving respiratory support compared with manual: A systematic review and meta‐analysis
Source: Pediatr Discov. 2024 May 22;2(4):e57. doi: 10.1002/pdi3.57 (PMC12118224; doi:10.1002/pdi3.57)

	automated			manual				Mean Difference	
**Study or Subgroup**	**Mean**	**SD**	**Total**	**Mean**	**SD**	**Total**	**Weight**	**IV, Fixed, 95% CI**	**Year**
Claure 2001	0.34	0.12	14	0.34	0.11	14	3.3%	0.00 [-0.09, 0.09]	2001
Claure 2011	0.32	0.12	32	0.37	0.19	32	3.9%	-0.05 [-0.13, 0.03]	2011
Wilinska 2015	0.32	0.07	15	0.33	0.09	15	7.2%	-0.01 [-0.07, 0.05]	2015
van Kaam 2015	0.31	0.08	40	0.3	0.09	40	17.2%	0.01 [-0.03, 0.05]	2015
Waitz 2015	0.32	0.15	15	0.3	0.12	15	2.5%	0.02 [-0.08, 0.12]	2015
Lal 2015	0.34	0.12	14	0.36	0.12	13	2.9%	-0.02 [-0.11, 0.07]	2015
van Kaam 2015	0.35	0.11	25	0.33	0.09	25	7.7%	0.02 [-0.04, 0.08]	2015
vd Heuvel 2018	0.25	0.05	41	0.27	0.05	41	51.1%	-0.02 [-0.04, 0.00]	2018
Gajdos 2019	0.31	0.1	12	0.29	0.09	12	4.1%	0.02 [-0.06, 0.10]	2019
Dijkman 2021	0.3	0.01	27	0.29	0.01	27		Not estimable	2021
**Total (95% CI)**			**208**			**207**	**100.0%**	**-0.01 [-0.02, 0.01]**	

Heterogeneity: Chi² = 5.09, df = 8 (P = 0.75); I² = 0%

Test for overall effect: Z = 1.13 (P = 0.26)

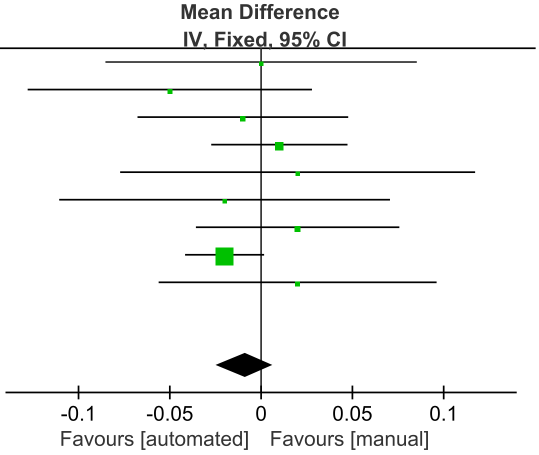

Supplement: Supplementary file 5 — Figure S4 [file PDI3-2-e57-s004.pdf]
