# Supplementary material for: The effectiveness of automated adjustment of inspired oxygen in preterm infants receiving respiratory support compared with manual: A systematic review and meta‐analysis
Source: Pediatr Discov. 2024 May 22;2(4):e57. doi: 10.1002/pdi3.57 (PMC12118224; doi:10.1002/pdi3.57)
Supplement: Supplementary file 9 — Figure S8 [file PDI3-2-e57-s007.pdf]

Author(s): Yihan Zhang, Yuxuan Du, Yuan Shi
Question: Automated compared to manual adjustments of inspired oxygen for preterm infants receiving respiratory support
Setting:
Bibliography: Refer to Reference.

Certainty assessment						№ of patients		Effect		Certainty	Importance
№ of studies	Study design	Risk of bias	Inconsistency	Indirectness	Imprecision	Other considerations	automated	manual adjustments of inspired oxygen	Relative (95% CI)	Absolute (95% CI)		
**The percentage of time within the target SpO2 range**												
16	randomised trials	not serious	serious[a]	not serious	not serious	none	388	387	-	MD **11.93 higher** (8.95 higher to 14.91 higher)	⨁⨁⨁◯ Moderate	IMPORTANT
**Percentage of time above the target SpO2 range**												
13	randomised trials	not serious	serious[a]	not serious	not serious	none	364	364	-	MD **7.15 lower** (10.41 lower to 3.9 lower)	⨁⨁⨁◯ Moderate	IMPORTANT
**Percentage of time below the target SpO2 range**												
13	randomised trials	not serious	serious[a]	not serious	not serious	none	364	364	-	MD **3.12 lower** (0.607 lower to 0.18 lower)	⨁⨁⨁◯ Moderate	IMPORTANT
**Percentage time of hypoxemia**												
10	randomised trials	not serious	serious[a]	not serious	not serious	none	303	303	-	MD **0.99 lower** (1.48 lower to 0.5 lower)	⨁⨁⨁◯ Moderate	IMPORTANT
**Manual FiO2 adjustments/hour**												
9	randomised trials	not serious	serious[a]	not serious	not serious	none	221	221	-	MD **2.89 lower** (4.37 lower to 1.41 lower)	⨁⨁⨁◯ Moderate	IMPORTANT
**Mean FiO2**												
10	randomised trials	not serious	not serious	not serious	serious[b]	none	208	207	-	MD **0.01 lower** (0.02 lower to 0.01 higher)	⨁⨁⨁◯ Moderate	IMPORTANT
**Mean SpO2**												
9	randomised trials	not serious	serious[a]	not serious	serious[b]	none	178	178	-	MD **0.15 lower** (0.57 lower to 0.27 higher)	⨁⨁◯◯ Low	IMPORTANT

CI: confidence interval; **MD:** mean difference

Explanations

a. Heterogeneity is large but explainable, so drop one level
b. 95% confidence interval CI over the line
